# Tagging the Expressed Protein with 6 Histidines: Rapid Cloning of an Amplicon with Three Options

**DOI:** 10.1371/journal.pone.0063922

**Published:** 2013-05-15

**Authors:** Manika Indrajit Singh, Vikas Jain

**Affiliations:** Microbiology and Molecular Biology Laboratory, Department of Biological Sciences, Indian Institute of Science Education and Research, Bhopal, India; University of Massachusetts Medical School, United States of America

## Abstract

We report the designing of three expression vectors that can be used for rapid cloning of any blunt-end DNA segment. Only a single set of oligonucleotides are required to perform the amplification of the target DNA and its cloning in all three vectors simultaneously. The DNA thus cloned can express a protein either with or without a hexa-histidine tag depending upon the vector used. The expression occurs from T7 promoter when transformed into *E. coli* BL21(DE3). Two of the three plasmids have been designed to provide the expressed protein with either N- or C-terminus 6 histidine amino acids in tandem. The third plasmid, however, does not add any tag to the expressed protein. The cloning is achieved quickly with the requirement of phosphorylation of PCR product without any restriction digestion. Additionally, the generated clones can be confirmed with a single step PCR reaction carried out from bacterial colonies (generally termed as “colony PCR”). We show the cloning, expression and purification of Green Fluorescent Protein (GFP) as proof-of-concept. Additionally, we also show the cloning and expression of four sigma factors from *Mycobacterium tuberculosis* further demonstrating the utility of the designed plasmids. We strongly believe that the vectors and the strategy that we have developed will facilitate the rapid cloning and expression of any gene in *E. coli* BL21(DE3) with or without a hexa-histidine tag.

## Introduction

Cloning of a gene is typically the first step in any molecular biology experiment. Insertion of a DNA segment in an expression vector normally requires PCR amplification of the target DNA followed by restriction digestion and ligation in the desired plasmid. T7 bacteriophage promoter based expression system is by far the most common system for protein expression in bacteria [1]–[3]. When transformed into *E. coli* BL21(DE3) cells, this promoter allows high level expression of the foreign gene upon addition of a gratuitous inducer, Isopropyl β-D-1-thiogalactopyranoside (IPTG), thereby resulting in the production of large amounts of protein [4]–[6]. Addition of IPTG in the culture medium leads to the expression of T7 RNA Polymerase from the lysogenized lambda DE3 that further results in the transcription from the T7 promoter present in the vector. Any foreign gene cloned downstream to this promoter is thus expressed [3], [4].

Several vectors are available commercially that allow the cloning of any gene of interest for expression and have been used extensively in literature [1], [2], [7], [8]. These plasmids allow the expression of foreign gene with or without a tag. Since a large number of biophysical and biochemical experiments require proteins to be present in their most purified form, they are generally expressed with an affinity tag for ease of purification [1], [2], [7]. Various tags such as the hexa-histidine tag (His_6_ tag), glutathione-S-transferase tag and chitin-binding domain tag etc. are available that allow single step purification of most proteins [2], [8]. The hexa-histidine tag provides a distinct advantage over others due to its usability in protein purifications under denaturing conditions (such as in the presence of urea and guanidine hydrochloride) also. This further helped in the development of methods for the on-column refolding of denatured proteins [9]–[13]. Although, in a rare instance, it has been shown that the addition of a histidine-tag in the protein promotes multimerization [14]. Cloning in these plasmids usually require the use of restriction enzyme sites that should not be present within the gene of interest. These sites are consequently introduced on the oligonucleotides at the desired location leading to the designing of longer primers for this purpose. The placement of the tag at the N- or the C-terminus of a protein is, by and large, decided based upon the choice of vector available at hand. However, repositioning of the same at a later stage becomes a difficult task, which requires either a careful selection of a distinct set of restriction enzyme sites to perform sub-cloning in another vector or a separate PCR experiment with newly designed primers. Furthermore, since the position of the tag has been shown to affect protein’s activity [15], it becomes necessary to reposition the tag. It therefore seems logical to prepare all likely constructs (both N- and C-terminus tagged as well as protein devoid of any tag) together. However, this, more often than not, requires PCR amplification of the gene with another set of oligonucleotides that harbor different restriction enzyme sites. With the combination of Gateway cloning and ligation independent cloning methods, it is now feasible to carry out such cloning experiments with ease; these strategies, nevertheless, require longer oligonucleotides with unique restriction enzyme sites [16]–[19].

In this manuscript, we report the designing and construction of three plasmids that will allow the expression of any foreign gene using the T7 expression system. All of these plasmids can incorporate any gene of interest, thereby avoiding restriction enzyme digestion of the PCR product. Two of the designed vectors will result in the production of recombinant proteins with a His_6_ tag at either N- or C-terminus. With the third vector, it is possible to obtain the expressed protein without any tag. It must be noted here that the amplification of the target DNA requires only a single set of primers for performing cloning in all three plasmids. Furthermore, we show the usability of these plasmids by carrying out the cloning, expression and purification of green fluorescent protein (GFP). We further show the utility of these plasmids by cloning four extracytoplasmic sigma factor genes from *Mycobacterium tuberculosis* and their expression.

It is thus possible to clone and express any foreign DNA in *E. coli* with or without hexa-histidine tag while avoiding repeated sub-cloning using different restriction enzymes.

## Materials and Methods

### Bacterial Strains, Plasmids and Growth Conditions

The pET21b and pET15b expression plasmids were purchased from Novagen. Chemically competent *E. coli* strain EXPRESS BL21(DE3) and XL1-blue were obtained from Lucigen and Stratagene respectively. *E. coli* bacterial strains were grown in Luria-Bertani medium (HiMedia), either as liquid culture with constant shaking at 200 rpm or on 1.5% agar plate, at 37°C. Cultures were always supplemented with 100 µg/ml Ampicillin unless otherwise specified. All molecular biological methods and the necessary precautionary measures were followed as described [20]. The plasmid pSK01-NCHS was kindly provided by Soumya Kamilla, IISER Bhopal, India.

### Reagents

Restriction endonucleases, Antarctic phosphatase and T4 Polynucleotide Kinase (PNK) were obtained from New England Biolabs (NEB) and were used following manufacturer’s instructions. T4 DNA ligase was obtained from Fermentas and used as suggested. Plasmid DNA purification and gel DNA extraction kits were purchased from Qiagen and Sigma respectively. 2-log DNA ladder was procured from NEB for DNA electrophoresis on agarose gels. PiNK Plus protein ladder was acquired from GeneDireX and was used as instructed in acrylamide gel electrophoresis experiments. All other reagents were purchased from Sigma.

### PCR and Site Directed Mutagenesis

PCR reactions were carried out using Phusion high fidelity DNA polymerase (NEB) as per the protocol provided by the supplier. Primers used in PCR and site-directed mutagenesis (SDM) experiments were procured from Macrogen (South Korea). To perform a colony PCR, a small amount of bacterial colony was re-suspended in 20 µl of MilliQ water and was heated at 100°C for 15 min. The sample was then spun at 13000 rpm for 5 min at room temperature (RT) and the resulting supernatant was used in the PCR reaction. SDM experiments were performed using primers listed in Table 1 by following multiple-site targeted mutagenesis method [21]. The PCR product was treated with 20 units of DpnI for 1 hour to remove the methylated parental strand [22] and was transformed into *E. coli* XL1blue cells. Positive clones were screened using restriction digestion of the isolated plasmid. Mutations were further confirmed by sequencing.

**Table 1:** Primers used in this study. SmaI site, wherever present, has been underlined. The forward primers for the cloning of genes start with ATG (boldfaced).

Primer	Sequence (5′–3′)
RACL-Site1	GATGGCTGCTGCCCCCGGGATATCTCCTTCTTAAAGTTAAAC
RACL-Site2	CGCGCGGCAGCCCCGGGAGCCCCAAGATCC
RACL-Site3	GTGGTGGTGGTGCCCGGGGTTCCAGGGCTGCGTG
RACL-Site4	GCACCACCACCACCCCGGGTGAGATCCGGCTGCTAACAAAGC
T7rev	GCTAGTTATTGCTCAGCGG
GFPFor-rapid	**ATG** AGCAAGGGCGAGGAGCTGTTC
GFPRev-rapid	CTTGTACAGCTCGTCCATGCCGAG
PETFOR	ATCGAGATCTCGATCCCGCGAAATTAATACG
RvSigBFor	**ATG** GCCGATGCACCCACAAGGGCCA
RvSigBRev	GCTGGCGTACGACCGCAGCCGATCC
RvSigDFor	**ATG** GTCGATCCGGGAGTTAGCCCG
RvSigDRev	CGCATAGTCACCTGCCGCAACAATCTCG
RvSigFFor	**ATG** ACGGCGCGCGCTGCCGGC
RvSigFRev	CTCCAACTGATCCCGTAGCCGTGCC
RvSigGFor	**ATG** CGCACATCGCCGATGCCCG
RvSigGRev	CAGCGAATCGGGCAGGCCGAATTTCG

### Cloning of GFP

Two primers, GFPFor-rapid and GFPRev-rapid (Table 1), were used to PCR amplify GFP gene from u-msfGFP vector (Addgene plasmid 29772; a kind gift from Scott Gradia, QB3 MacroLab, University of California Berkeley). The amplicon was phosphorylated using T4 PNK (NEB) and the enzyme was inactivated following supplier’s instructions. The amplified DNA product was further purified using ethanol precipitation. The vector backbone was prepared by digesting the pMS-QS vectors (Table 2; described later) with SmaI. The vectors were dephosphorylated using Antarctic phosphatase and purified by ethanol precipitation. The ligation of the amplified DNA product and the vectors (in the ratio of 3∶1) was carried out following manufacturer’s instructions, using T4 DNA ligase and the mixture was transformed in XL1blue cells. Screening for the positive clones was attempted by the colony PCR using PETFOR and GFPRev-rapid primers (Table 1). Clones were confirmed by sequencing.

**Table 2:** Plasmids used in this study. All the plasmids carry Ampicillin resistance marker and the cloned gene expresses from a T7 promoter, when an appropriate host is used.

Plasmids	Size (kbp)	Description	Source
pSK01-NCHS	5.8	∼500 bp *orf* sandwiched between N- & C-ter His_6_ tag	Kind gift of Soumya Kamilla, IISER Bhopal
pMS-QS-NOHS	5.3	No His_6_ tag is expressed along with the gene	This study
pMS-QS-CHS	5.3	C-ter His_6_ tag is expressed along with the gene	This study
pMS-QS-NHS	5.4	N-ter His_6_ tag is expressed along with the gene	This study
pMS-QS-NOHGFP	6.0	GFP expression without any tag	This study
pMS-QS-CHGFP	6.0	GFP expression with His_6_ tag at its C-ter	This study
pMS-QS-NHGFP	6.1	GFP expression with His_6_ tag at its N-ter	This study
pMS-QS-NOHSigB	6.3	H37Rv Sigma factor B expression without any tag	This study
pMS-QS-CHSigB	6.3	H37Rv Sigma factor B expression with His_6_ tag at its C-ter	This study
pMS-QS-NHSigB	6.4	H37Rv Sigma factor B expression with His_6_ tag at its N-ter	This study
pMS-QS-NOHSigD	5.9	H37Rv Sigma factor D expression without any tag	This study
pMS-QS-CHSigD	5.9	H37Rv Sigma factor D expression with His_6_ tag at its C-ter	This study
pMS-QS-NHSigD	6.0	H37Rv Sigma factor D expression with His_6_ tag at its N-ter	This study
pMS-QS-NOHSigF	6.1	H37Rv Sigma factor F expression without any tag	This study
pMS-QS-CHSigF	6.1	H37Rv Sigma factor F expression with His_6_ tag at its C-ter	This study
pMS-QS-NHSigF	6.2	H37Rv Sigma factor F expression with His_6_ tag at its N-ter	This study
pMS-QS-NOHSigG	6.4	H37Rv Sigma factor G expression without any tag	This study
pMS-QS-CHSigG	6.4	H37Rv Sigma factor G expression with His_6_ tag at its C-ter	This study
pMS-QS-NHSigG	6.5	H37Rv Sigma factor G expression with His_6_ tag at its N-ter	This study

### GFP Expression and Zymography

The plasmids carrying GFP gene (Table 2) were transformed in *E. coli* BL21(DE3) cells. Each clone was grown at 37°C at 200 rpm in 5 ml of LB medium. At an optical density at 600 nm (OD_600_) ∼0.8, 0.5 ml of culture was withdrawn and 1 mM IPTG (final concentration) was added to the remaining medium for protein expression. The cultures were allowed to grow further for 3 hours. The bacteria in all these cases were harvested by centrifugation and 50 µl of 8 M urea and 10 µl of 5X SDS gel loading buffer [23] was added to each of them. The cells were lysed by heating at 100°C for 5 min and 8 µl of each of the samples were run on 12% SDS-Polyacrylamide gel electrophoresis (PAGE). After carrying out the electrophoresis, the gel was incubated in 1% Triton X-100 for 2 hours and was then photographed, to obtain a zymogram, with epi-illumination at 480 nm and SYBR Gold filter (485–655 nm) in a UVP gel documentation system (UVP, LLC). Later, the gel was fixed in 10% acetic acid, 1% trichloroacetic acid (TCA) and 40% methanol solution in water for 1 hour and was then stained with Coomassie brilliant blue dye R-250 to visualize protein bands.

### Purification of GFP

The clones expressing GFP along with His_6_ tag (Table 2) were grown at 37°C at 200 rpm in 250 ml of LB medium. The protein was expressed by the addition of 1 mM IPTG when the culture reached an OD_600_ ∼0.8. The induction was continued for 3 hours. The cells were then harvested by centrifugation at 4°C and resuspended in the lysis buffer (50 mM sodium phosphate buffer, 500 mM NaCl, 5 mM β-mercaptoethanol, 10 mM imidazole, 5% glycerol) supplemented with 10 µg/ml lysozyme (chicken egg white lysozyme; Sigma) and incubated on ice for 30 min. Cells were then lysed by sonication and the lysate was cleared by centrifugation. The supernatant was incubated with 250 µl bed volume of Ni-NTA (Nickel-Nitrilotriacetic acid) agarose (Qiagen), pre-equilibrated with the lysis buffer, for 1 hour with constant mixing at 4°C. The matrix was collected and washed with wash buffer (50 mM sodium phosphate buffer, 1 M NaCl, 5 mM β-mercaptoethanol, 20 mM imidazole, 5% glycerol). Elution of protein was then carried out in 10 column volumes of elution buffer (50 mM sodium phosphate buffer, 500 mM NaCl, 5 mM β-mercaptoethanol, 200 mM imidazole, 5% glycerol) and the collected elution fractions were analyzed on a 12% SDS-PAGE [23].

### Cloning of *Mycobacterium tuberculosis* Sigma Factors and their Expression Analysis

Four extracytoplasmic sigma factors (SigB, D, F & G) from *M. tuberculosis* were cloned in the designed pMS-QS vectors. The genes were PCR amplified using the *M. tuberculosis* H37Rv genomic DNA as template (kindly provided by AstraZeneca, Bangalore) and the primers listed in Table 1. The PCR products were extracted from the agarose gel and were ligated into the linear pMS-QS vectors. The resultant products were transformed in *E. coli* XL1Blue and the positive clones were identified by carrying out colony PCR. All the clones were further confirmed by sequencing. The plasmids thus constructed (as listed in Table 2) were transformed in *E. coli* BL21(DE3) for protein expression as described previously.

## Results

In the present manuscript, we report the designing and construction of pMS “quick series” vectors. These vectors contain a restriction enzyme site that blunt cuts the vector for cloning of the PCR generated DNA fragments. The vector carries a T7 promoter and a ribosome binding site upstream of the cloning site with appropriate addition of the codons for six histidine amino acids, as discussed below.

### Construction of pMS-QS Vectors

**Figure 1:**
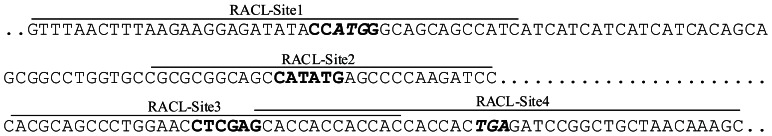
Sequence of a region of pSK01-NCHS showing the SDM primer binding sites. Italicized letters represent the start and stop codon. Bold faced letters depict restriction enzyme sites, NcoI, NdeI and XhoI, which were mutated to SmaI.

**Figure 2:**
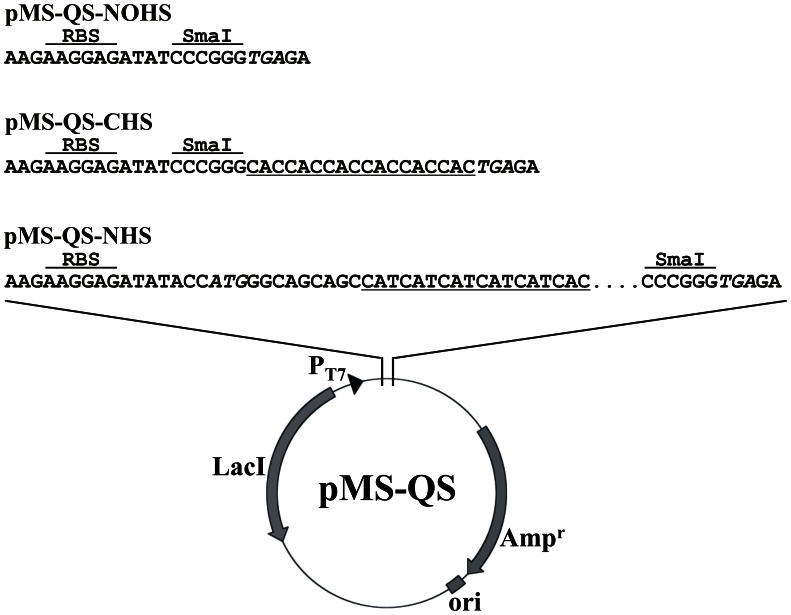
Diagrammatic representation of the three pMS “quick series” vectors. Cloning region of all the three vectors as achieved after SDM has been shown. These vectors have DNA insertion site (SmaI site) downstream to ribosome binding site (RBS). The C-ter and N-ter histidine tags are underlined in case of pMS-QS-CHS and pMS-QS-NHS respectively. The dotted line in pMS-QS-NHS represents the thrombin cleavage site originally present in pET15b vector.

The vector pSK01-NCHS (kindly provided by Soumya Kamilla) was constructed by cloning a 500 bp DNA fragment between NdeI and XhoI sites of pET21b followed by cloning of an NdeI/EcoRV fragment, obtained from pET15b, in this vector at the same sites. This construct was prepared for a different study and will be discussed elsewhere. The resulting plasmid provided nucleotides coding for six histidine amino acids at both ends of the cloned gene (data not shown). The pMS “quick series” expression vectors were constructed by carrying out site-directed mutagenesis to introduce SmaI sites in pSK01-NCHS at different locations (Fig. 1) using the primers listed in Table 1. Mutagenesis was carried out following the multiple-site targeted mutagenesis method [21]. The vector, pMS-QS-NOH-Ins, was prepared by replacing the NcoI site and the six base pairs upstream of the stop codon with SmaI using RACL-Site1, RACL-Site4 and T7 rev primers. The resulting plasmid lost the translation initiation site ATG but retained the stop codon. Similarly, primers RACL-Site2, RACL-Site4 along with T7 rev were used to introduce SmaI sites at NdeI and six base pair upstream of translation stop codon to construct pMS-QS-NHS-Ins. RACL-Site1 and RACL-Site3 primers were sufficient to replace NcoI and XhoI sites in pSK01-NCHS to SmaI yielding pMS-QS-CHS-Ins. Clones obtained were screened by SmaI digestion and were further confirmed by sequencing. These vectors were then digested with SmaI to remove the cloned 500 bp region. The vector backbone was gel extracted, self-ligated and transformed in *E. coli* XL1Blue cells. The process yielded three pMS-QS vectors viz. pMS-QS-NOHS, -CHS and -NHS (Fig. 2) that were further confirmed by sequencing. These plasmids are available upon request.

### Cloning can be Rapidly Carried out in pMS-QS Vectors

**Figure 3:**
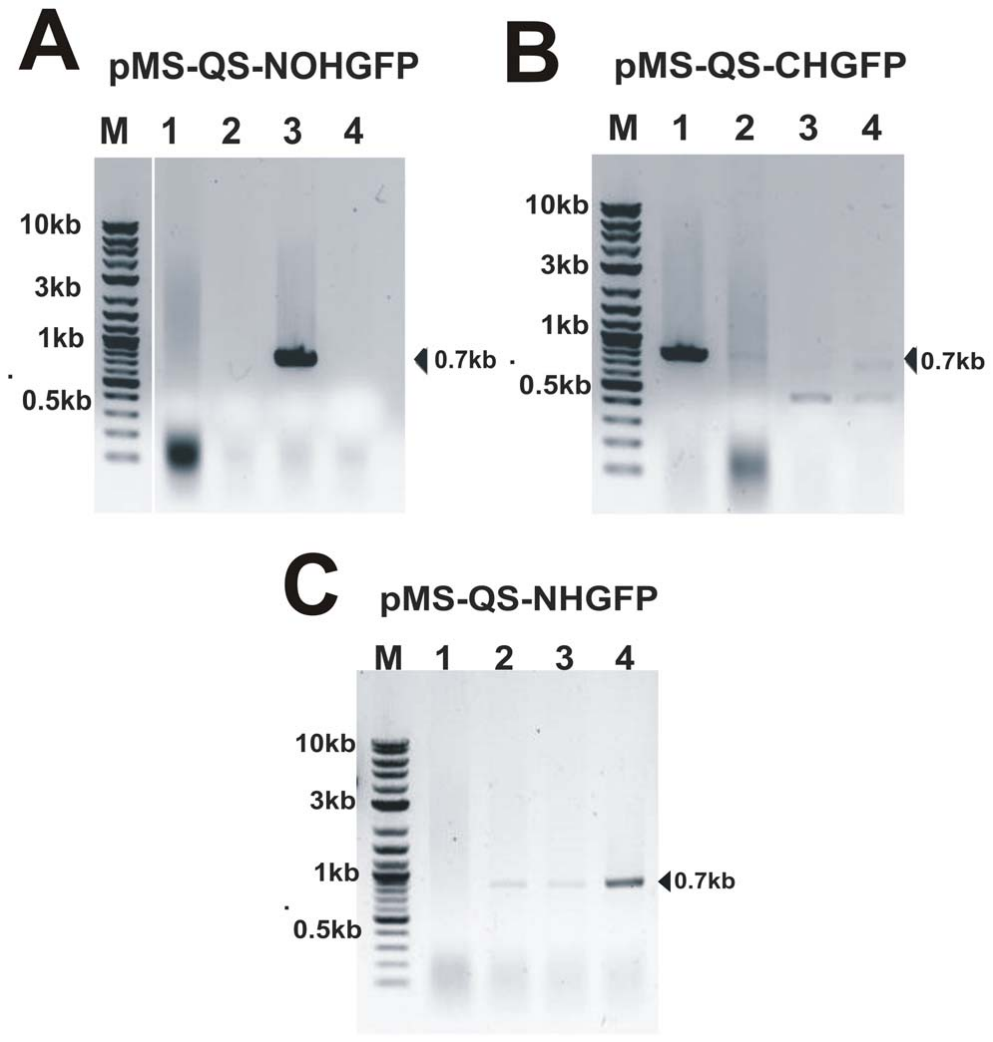
Screening of GFP positive clones using colony PCR. Agarose gel images after colony PCR showing, (A) clone-3 to be positive in pMS-QS-NOHGFP; (B) clone-1 to be positive in pMS-QS-CHGFP and (C) clones-2, 3 & 4 to be positive in pMS-QS-NHGFP. Vector specific forward primer (PETFor) and gene specific reverse primer (GFPRev-rapid) were used to carry out colony PCR in order to confirm the insertion as well as the orientation of the GFP gene in plasmid.

To show the efficacy of the designed vectors, we carried out the cloning of GFP gene in these vectors. A set of primers, GFPFor-rapid and GFPRev-rapid, was used to PCR amplify the GFP gene from u-msfGFP vector (Addgene plasmid 29772; a kind gift from Scott Gradia, QB3 MacroLab, University of California Berkeley). The forward primer, GFPFor-rapid carried the translation start site ATG whereas the stop codon was avoided in the reverse primer, GFPRev-rapid. The amplicon was phosphorylated and ligated in all the three SmaI-linearized and dephosphorylated pMS-QS vectors. The ligation mix was transformed in XL1blue cells. Since a visual examination for the positive clones (such as blue-white selection) is not possible in this case, the positive clones (with vector ligated to an insert in correct orientation) were identified by carrying out colony PCR (Fig. 3). The clones that appeared positive following this method were further confirmed by sequencing. Clones thus generated (Table 2) were expected to express GFP with either no histidine tag (pMS-QS-NOHGFP) or with an N-terminal (pMS-QS-NHGFP) or a C-terminal (pMS-QS-CHGFP) hexa-histidine tag. The utility of the designed vectors was further assessed by attempting the cloning of four extracytoplasmic sigma factors of *M. tuberculosis* H37Rv viz. SigB, SigD, SigF and SigG. These genes were PCR amplified from the genomic DNA of *M. tuberculosis* and were cloned directly in the designed pMS-QS expression vectors (Table 2).

### Genes Cloned in pMS-QS Vectors can be Expressed in BL21(DE3) Cells

**Figure 4:**
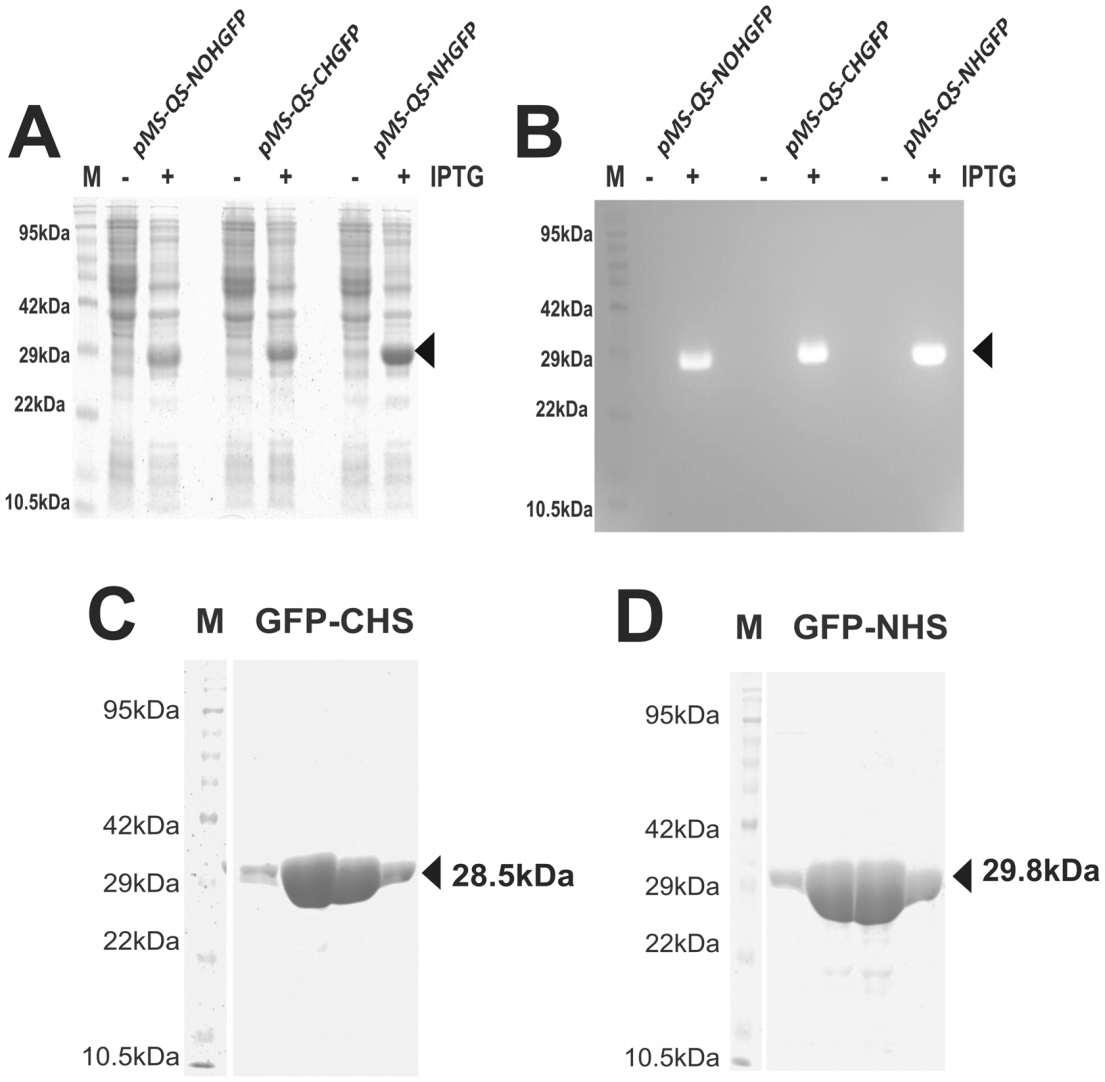
GFP expression from pMS-QS vectors. (A) GFP induction profile with all the three vectors. Both uninduced (−IPTG) and induced (+IPTG) cultures have been compared. Arrow depicts the induced GFP expression. (B) Zymogram of A that was imaged using 480 nm light and SYBR Gold filter. The arrow represents the folded GFP protein in polyacrylamide gel that fluoresces upon excitation with 480 nm light. Pre-stained protein marker was used in order to make it appear on zymogram. Purification of GFP protein using Ni-NTA matrix was done after overexpressing the protein in the bacterial cells having pMS-QS-CHGFP vector expressing C-ter GFP (C) and pMS-QS-NHGFP vector expressing N-ter GFP (D). The arrow shows the purified protein band. Only elutions were loaded for analysis.

**Figure 5:**
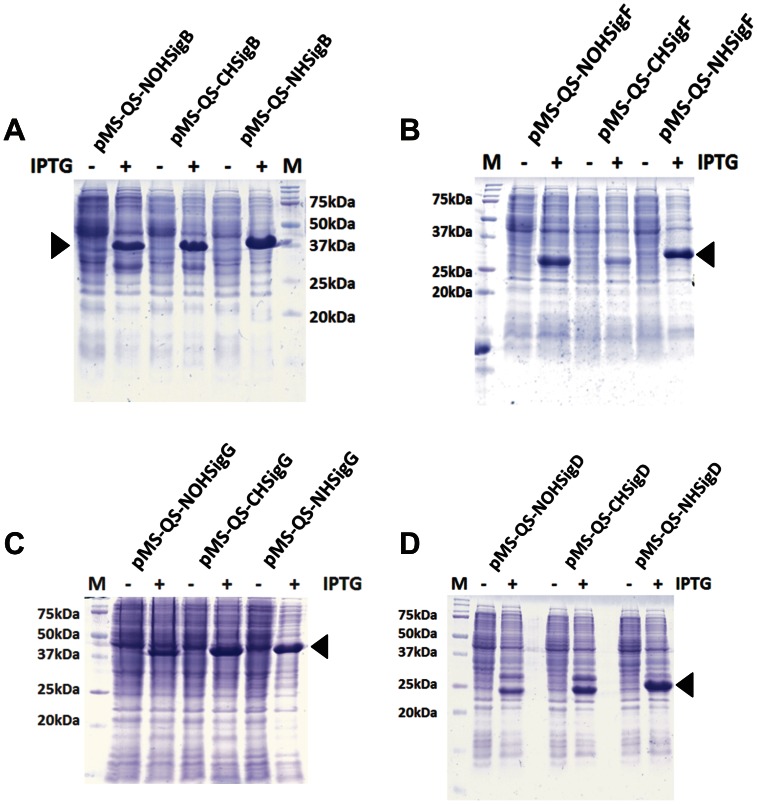
Expression analysis of *M. tuberculosis* sigma factors. All sigma factors cloned in pMS-QS vectors were expressed in *E. coli* BL21(DE3) and were analyzed on a 15% SDS-PAGE gel. The gel images show protein profiles in the presence (+) and absence (−) of IPTG. Arrow points to the protein produced upon addition of IPTG only. Panels A, B, C and D correspond to SigB, SigF, SigG and SigD respectively. “M” represents protein molecular weight marker.

We attempted the expression of GFP cloned in pMS “quick series” vectors in *E. coli* BL21(DE3). The induction of the bacterial culture showed the production of a protein of expected size (Fig. 4A & B). The three proteins were shown to be functional by carrying out the zymography (Fig. 4B). The purification of the GFP was carried out only in the case of tagged protein using immobilized metal ion affinity chromatography (IMAC) [24]. Both the N-terminus and the C-terminus tagged GFP could be purified successfully (Fig. 4C & D) using Ni-NTA purification method suggesting that the pMS-QS vectors can be used for the rapid cloning, expression and the purification of the proteins. The expression of the cloned sigma factors from *M. tuberculosis* in *E. coli* BL21(DE3) was carried out following the protocol as described above for GFP. The proteins were produced by the designed clones upon addition of IPTG in the culture medium (Fig. 5) thus demonstrating the utility of these plasmids in rapid cloning and expression of genes.

## Discussion

**Figure 6:**
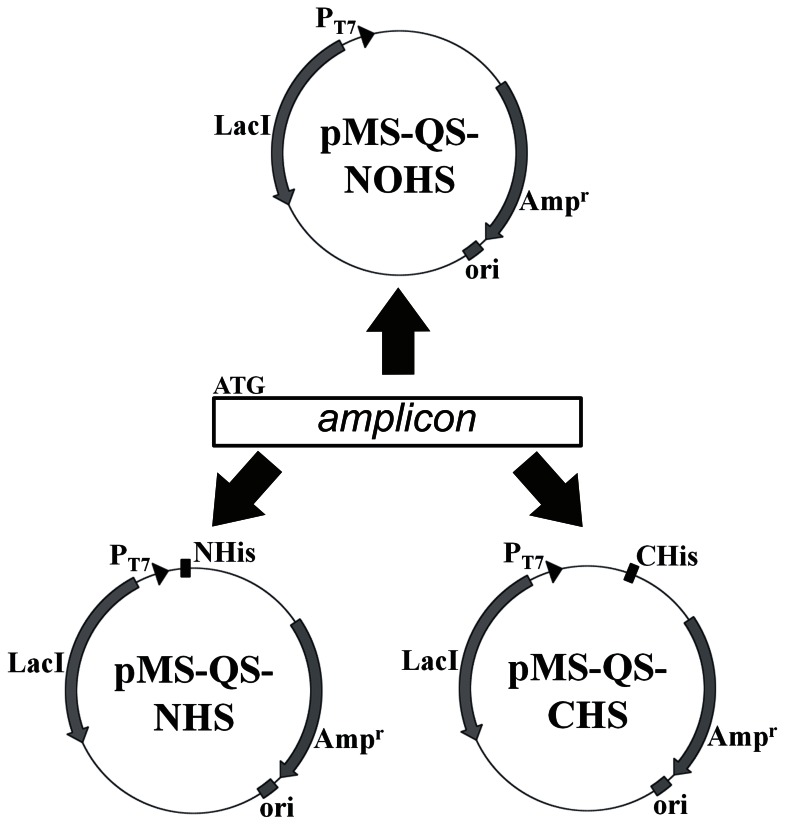
Diagrammatic representation of the cloning of a PCR amplified DNA segment into the three vectors. The amplified product should contain the ATG. The blunt-end DNA segment can be cloned in all the three vectors.

We present here the designing of three expression vectors that can be used for the cloning and expression of any blunt-end DNA fragment. Together, these vectors provide an efficient and rapid tool to not only express any gene in *E. coli* along with a His_6_ tag at either of the termini of the protein, but also allow the expression of the protein without any tag. We wish to emphasize that the cloning in all three vectors can be carried out in parallel with one DNA product prepared by using one set of oligonucleotides (Fig. 6). Furthermore, only short primers without any restriction enzyme sites are needed in order to clone a gene. Thus, the digestion of the amplified gene product is not required when these plasmids are used for cloning purpose thereby saving time and enzyme costs. Additionally, a single step colony PCR reaction is sufficient to screen for positive clone with gene inserted into the vector in correct orientation. Cloning in these vectors does pose a condition on the designing of the primers. While the forward primer must start with an ATG trinucleotide, the reverse primer should not contain the stop codon. The former is relaxed only when an N-ter His_6_ tag is desired whereas the latter becomes stringent when the C-ter hexa-histidine tag is a required feature. The utility of the designed constructs has been shown by carrying out the cloning and expression of GFP and four sigma factors from *Mycobacterium tuberculosis*. We, therefore, strongly believe that the system developed here will aid tremendously in rapid cloning of genes, expression and purification from various systems.

The use of Taq DNA polymerase for PCR experiments is not strongly recommended for two reasons. Taq polymerase is known not to have a very high fidelity and is, therefore, generally avoided for the PCR amplification of DNA especially where the downstream application requires gene expression [20]. It has also been shown that Taq DNA polymerase adds an additional base at the end of the amplicon [25]. It is conceivable, therefore, that the “extra” nucleotide will either not allow ligation or may lead to a frame-shift before the stop codon. Although, we do hypothesize that the addition of one nucleotide will not affect the translation initiation due to the intact start codon if the ligation and transformation do yield a positive clone, such an event will not lead to any expression of the recombinant protein due to the frame-shift immediately after the N-ter His_6_ tag.

### Conclusion

We believe that the vectors designed in this study, collectively, will facilitate rapid cloning of any gene without using a restriction enzyme. The PCR amplicon generated with a single set of primers can be directly ligated in any of these vectors to yield the desired tag in the expressed protein. The procedure will help in avoiding additional sub-cloning when a tag at a different terminus in the protein is desired. The positive clone can be easily screened using colony PCR method. We further wish to add that these vectors may find their suitability in the experiments involving cDNA library preparation in bacteria. However, experimental evidence, in this regard, is currently unavailable.
